# Paravalvular Leak Closure After Transcatheter Tricuspid Valve-in-Ring Implantation: A Case Report

**DOI:** 10.7759/cureus.61059

**Published:** 2024-05-25

**Authors:** Wassim Assaad, Dounia Iskandarani, Walid Gharzuddine, Fadi Sawaya

**Affiliations:** 1 Cardiology, American University of Beirut, Beirut, LBN; 2 Cardiology, American University of Beirut Medical Center, Beirut, LBN

**Keywords:** transcatheter valve-in-ring implantation, tricuspid valve annuloplasty, right sided heart failure, tricuspid valve, paravalvular leak closure

## Abstract

Transcatheter tricuspid valve intervention (TTVI) has emerged as a promising alternative for patients with severe tricuspid regurgitation who are deemed high-risk for surgery. With advancements in device design and delivery systems, TTVI has shown promising outcomes in reducing tricuspid regurgitation severity and improving symptoms in selected patients. Paravalvular leaks (PVLs) are one of the most common complications faced, which can significantly contribute to patients’ morbidity and mortality. Percutaneous PVL closure represents a minimally invasive approach to address this issue, but its efficacy and safety in the context of transcatheter tricuspid valve-in-ring implantation require further elucidation.

We describe the case of a 44-year-old lady with a history of rheumatic valve disease status post-tricuspid valve annuloplasty with an incomplete ring who presented to cardiology clinics with symptomatic torrential tricuspid regurgitation. Due to the high risk of surgical reintervention secondary to severe right ventricular (RV) failure, she was denied surgical intervention. Therefore, she underwent transcatheter tricuspid valve-in-ring (TVIR) implantation with a 26 mm MyVal (Meril Life Sciences Pvt Ltd., Vapi, GJ, IND), which was complicated by a residual severe tricuspid paravalvular regurgitation. The defect was subsequently closed by a dedicated Occlutech PVL device (Occlutech, Helsingborg, SWE) measuring 18 mm x 10 mm. Post which, the patient had trivial tricuspid regurgitation and significant improvement in signs and symptoms with subsequent follow-up.

## Introduction

Tricuspid regurgitation (TR) represents a complex and often underappreciated pathology of the cardiovascular system. While historically overshadowed by left-sided valvular diseases, recent advancements in imaging techniques and clinical awareness have shed some new light on the significance of TR. Tricuspid regurgitation is common and is reported in more than 80% of patients undergoing routine echocardiography, with prevalence increasing with advancing age [1,2]. While mild or no regurgitation is a common benign finding, moderate-to-severe TR is associated with a worse prognosis, independent of ventricular function and pulmonary artery systolic pressure [3,4].

The latest American Heart Association (AHA) guidelines recommend surgical intervention for progressive or severe TR in symptomatic patients with signs of right-sided heart failure, undergoing left-heart surgery, or tricuspid valve (TV) annular dilation >40 mm in diastole [5]. However, durability remains to be affected by the presence of poor LV function, pacemaker wire, an initial high grade of regurgitation, and repair type [6,7]. Mortality rates are high in patients who undergo redo surgery for the TV after failure of TV repair [6,8]. To mitigate the increasing risks, transcatheter tricuspid valve-in-valve or tricuspid valve-in-ring (TVIR) implantation emerges as a promising tool. However, transcatheter TVIR implantation presents a unique challenge as most of the rings used are rigid and incomplete, which increases the risk of paravalvular regurgitation [9].

Here, we describe the case of a 44-year-old lady with a history of TV annuloplasty for severe TR presenting to the cardiology clinics with dyspnea on exertion, abdominal distension, and lower limb edema. Echocardiography showed a dilated right ventricle, severely impaired systolic function, and torrential TR. Surgical intervention was refused by two surgeons given the high risk of mortality associated with reintervention secondary to severe right ventricular (RV) failure. We opted for a transcatheter TVIR implantation, which was complicated by residual severe TR at the site of the incomplete ring. This was successfully closed with a paravalvular leak (PVL) device plug with residual trivial regurgitation.

## Case presentation

A 44-year-old lady with a history of severe TR secondary to rheumatic TV disease status post annuloplasty with an incomplete 26 mm prosthetic rigid ring (Contour 3D, Medtronic, Minneapolis, MN, USA) 10 years ago presented to the cardiology clinics with progressively worsening dyspnea on exertion, abdominal distention, and lower limb edema. Her symptoms have been present for at least one year, with repetitive admissions for diuresis and paracentesis. Transesophageal echocardiography (TEE) showed a dilated right ventricle, severely impaired systolic function, and torrential TR due to persistent mal-coaptation of leaflets around 9 mm. Despite her worsening symptoms and repetitive hospital admissions, she was denied surgical intervention by two different surgeons due to the high risk of mortality associated with redo TV surgery in the presence of severe RV dysfunction.

**Figure 1:**
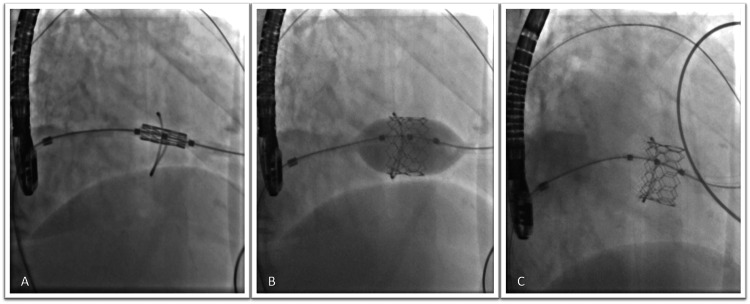
Fluoroscopic images show the positioning of the 26 MyVal transcatheter heart valve via a transcutaneous approach (A), its balloon dilation (B), and the final result (C).

**Figure 2:**
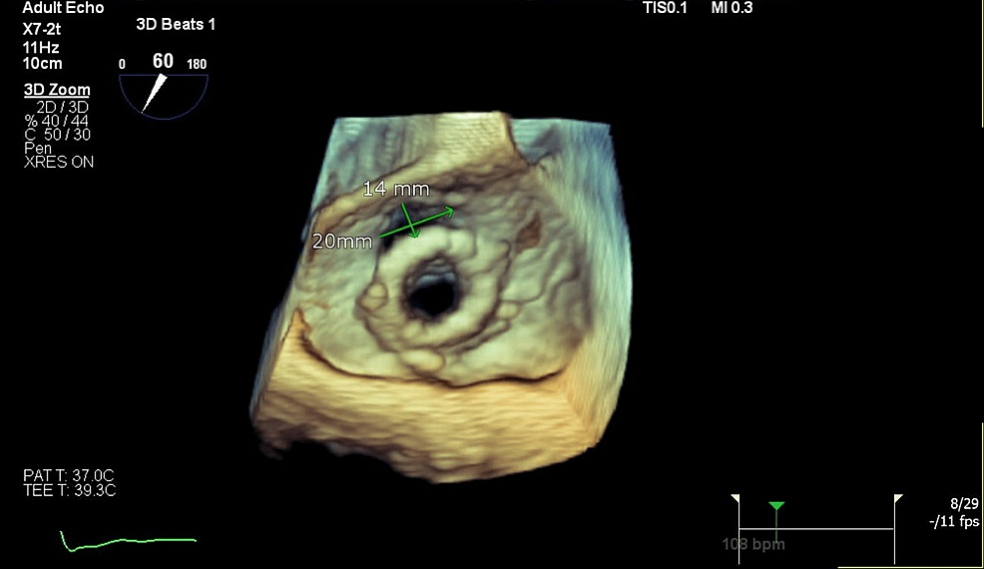
The 3D TEE shows the PVL (20 x 14 mm) defect around the bioprosthetic valve causing severe TR. TEE: Transesophageal echocardiography, PVL: Paravalvular leak, TR: Tricuspid regurgitation

The patient’s symptoms were initially treated with aggressive diuresis and abdominal paracentesis, with the successful removal of 15 L of fluid. Despite the unfavorable anatomy of the ring, it was decided that transcatheter TVIR implantation was the only viable option. Under conscious sedation and via transfemoral access, the patient underwent a percutaneous transcatheter TVIR with a 26 mm MyVal (Meril Life Sciences Pvt Ltd., Vapi, GJ, IND) (Figure 1), which was delivered via an Amplatz extra stiff wire (Boston Scientific, Marlborough, MA, USA). However, the TEE revealed a residual PVL, resulting in persistent severe TR at the incomplete portion of the ring on the septal side (Figure 2, Videos 1-2).

**Video 1:** The 2D TEE of the PVL on color Doppler. TEE: Transesophageal echocardiography, PVL: Paravalvular leak

**Video 2:** The 3D TEE of the PVL defect with regurgitation as seen by color Doppler imaging. TEE: Transesophageal echocardiography, PVL: Paravalvular leak

**Figure 3:**
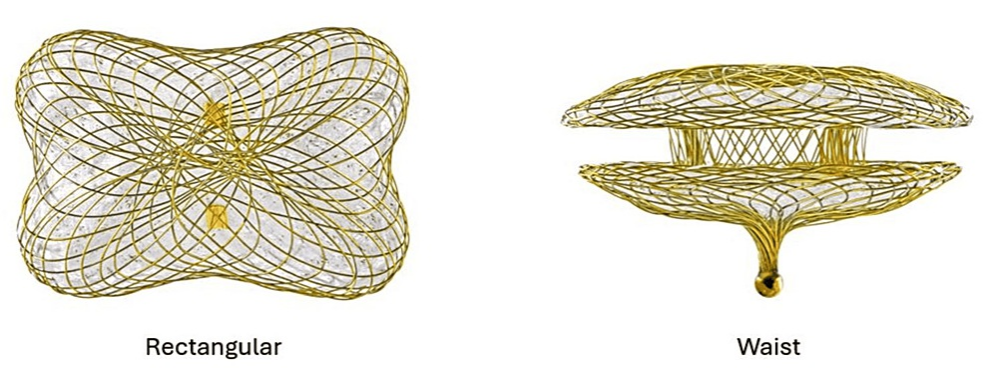
The Occlutech PVL device (18 mm x 10 mm) that was used to close the defect. PVL: Paravalvular leak Reproduced with permission from Mr. Rawand Fredriksson, Marketing Manager at Occlutech International AB, Helsingborg, Sweden.

We attempted to close the PVL using ventricular septal defect (VSD) plugs measuring 14 mm x 5 mm, the largest plug available in our lab at that time, but the gap was larger than expected. Therefore, we decided to reschedule the closure until we had the appropriate size. Two weeks later, the PVL was scheduled for closure under general anesthesia from a right femoral access. An Agilis catheter (Abbott, Green Oaks, IL, USA) mounted on a JR 4.0 (Medtronic) guiding catheter was used to guide the Terumo wire (Terumo Medical Corp., Tokyo, Japan) through the PVL. A dedicated large rectangular PVL device plug (Occlutech) measuring 18 mm x 10 mm (Figure 3, Videos 3-5) was used to close the leak, reducing the regurgitation from severe to trivial (Videos 6-7).

**Video 3:** Fluoroscopic image shows the deployment of an 18 mm x 10 mm plug to close the large PVL defect. PVL: Paravalvular leak

**Video 4:** The 2D TEE view of the deployment of the plug at the site of the defect. TEE: Transesophageal echocardiography

**Video 5:** The 3D TEE view of the deployment of the plug at the site of the defect. TEE: Transesophageal echocardiography

**Video 6:** The 2D TEE view with color Doppler shows the deployment of the plug at the site of the defect. TEE: Transesophageal echocardiography

**Video 7:** The 3D TEE view with color Doppler shows the deployment of the plug at the site of the defect. TEE: Transesophageal echocardiography

The patient had a transient atrioventricular (AV) block after the procedure, which resolved in less than 24 hours without any intervention. The patient was discharged the following day. Over three weeks, the patient lost almost 33 kg of weight, which was mainly related to the loss of fluids, accompanied by major improvements in her symptoms from New York Heart Association (NYHA) IV to NYHA II. After six months, the patient came for a follow-up visit, where she was assessed to have no new symptoms. Repeat echocardiography showed unchanged trace central TV regurgitation with persistent, severely impaired RV function.

## Discussion

Transcatheter innovations remain the perfect solution for patients with symptomatic recurrence of severe TR deemed at high risk of mortality for redo surgery. Despite the off-label indication of using transcatheter technologies over the TV, there are several case reports of successful TVIR implantation [10-13]. However, PVL remains an inevitable complication that may be more severe in some cases compared to others, depending on the position of the newly implanted valve and the type of ring used in the previous repair. In an international multicenter registry for successful off-label TVIR (n = 20), PVL was reported in 75% of patients, with the majority having trivial to mild regurgitation. A total of six patients required occlusive devices to close the PVL at the index procedure or on subsequent follow-up [11]. Paravalvular leaks are more likely to be seen with the use of rigid or semi-rigid incomplete rings or bands, which are most commonly used now to minimize the risks to the conduction system. The most common site is usually at the incomplete portion of the ring towards the septum [11], similar to our patient.

Data regarding PVL closure post-TVIR is limited to some case reports where vascular plugs or VSD occluders were used [10-13]. Here, we demonstrated the successful closure of a large PVL using a dedicated rectangular Occlutech PVL device measuring 18 mm x 10 mm. The key to a successful closure is adequate sizing of the defect, which can be done using 3D TEE or a CT scan. Our patient developed a transient asymptomatic complete AV block after a few hours from the procedure. This is most likely related to local edema at the location of the deployed PVL device towards the septum in proximity to the AV node. However, the heart block resolved in less than 24 hours without any intervention. There is no data in the literature regarding the best time to close a PVL. The decision is case-dependent, taking into consideration the severity of the leak, the size of the defect, the stability of nearby tissue, including the TV, the risk of complication, and the availability of an appropriate PVL occluding device size. In our case, we opted to delay the procedure until we had the right size to ensure adequate closure and minimize complications.

## Conclusions

A TVIR implantation remains a hopeful solution for patients with a symptomatic recurrence of severe TR after a previous annuloplasty or surgery. However, despite adequate alignment and sizing of the valve, the PVL remains an important complication of such a procedure. The main reason behind that is most likely related to the unfavorable anatomy of the incomplete ring, which leaves a gap for a PVL. We described the successful closure of such a PVL with a dedicated rectangular Occlutech device, with a significant reduction in regurgitation from severe to trivial. The key to success was deploying the right size of the PVL occluding device after adequate sizing of the PVL orifice using TEE 2D and 3D imaging. Careful attention should be paid to conduction disturbances that might occur post-procedure, given the critical location of most PVLs towards the AV node.
